# Supplementary material for: Application of FTIR Spectroscopy to Detect Changes in Skeletal Muscle Composition Due to Obesity with Insulin Resistance and STZ-Induced Diabetes
Source: Int J Mol Sci. 2022 Oct 18;23(20):12498. doi: 10.3390/ijms232012498 (PMC9603871; doi:10.3390/ijms232012498)
Supplement: Supplementary file 1 [file ijms-23-12498-s001.zip › ijms-1951340-supplementary.pdf]

Supplementary Material—SM

Application of FTIR Spectroscopy to Detect Changes in Skeletal Muscle Composition Due to Obesity with Insulin Resistance and STZ-Induced Diabetes

Barbara Zupančič [1,†], Nejc Umek [2,*,†], Chiedozie Kenneth Ugwoke [2], Erika Cvetko [2], Simon Horvat [3] and Jože Grdadolnik [1,*]

[1] Laboratory for Molecular Structural Dynamics, Theory Department, National Institute of Chemistry, 1000 Ljubljana, Slovenia
[2] Institute of Anatomy, Faculty of Medicine, University of Ljubljana, 1000 Ljubljana, Slovenia
[3] Chair for Genetics, Biotechnology and Immunology, Biotechnical Faculty, University of Ljubljana, 1230 Domžale, Slovenia
* Correspondence: nejc.umek@mf.uni-lj.si (N.U.); joze.grdadolnik@ki.si (J.G.)
† These authors contributed equally to this work.

Multivariate curve resolution (MCR) methods in vibrational spectroscopy

Compared to other factor analysis methods, multivariate curve resolution (MCR) methods provide the vector profiles with direct physical meaning and do not just explain the data variance [1]. They aim to obtain the profiles of the constituents for various types of mixture analysis problems where little or no knowledge of the profiles of the pure components is available [1]. MCR has also been established in vibrational spectroscopy as one of the most powerful and promising approaches among the various spectral decomposition strategies [2,3]. Essentially, it is a difference spectroscopy in which the vibrational spectrum is considered as an additive mixture of the unknown, non-negative and linearly independent spectral components [2]. MCR methods focus on the extraction of the spectral components in a mixture system through a bilinear model decomposition written as follows:

$$D = CS^T + E, \tag{S1}$$

where D is the matrix of experimental data (the number of rows corresponds to the number of spectra of the different mixtures and the columns to the different wavelengths). The S^T matrix from the decomposition contains information about the spectral components (the transposition of the matrix indicates that the spectra are in the rows), while the C matrix stores the concentration of each of the spectral components from S^T. The matrix E contains the data variance not explained by the components from the decomposition.

One of the recently used MCR methods to solve the MCR bilinear model of Equation (S1) is the alternating least squares (MCR-ALS) optimization under constraints. In this approach, the number of components that can be estimated by principal component analysis (PCA) or singular value decomposition (SVD), the initial estimates of the concentrations, and the choice of constraints (non-negativity, unimodality, closure, etc.) that reduce the ambiguity of the MCR solutions must be considered [1].

In our analysis of skeletal muscle tissue composition, we used the MCR-ALS method to decompose the ATR-FTIR spectra in the range 4000 cm^{-1} to 600 cm^{-1}. The spectra were obtained by processing the

recorded interferograms with a resolution of 2 cm^{-1} and 64 scans averaged for each spectrum using the OPUS software. The same software was used to perform atmospheric water and CO_2 compensation, baseline correction, and min-max normalization (so that the minimum is 0 and the maximum absorbance of the highest peak is 2) for each spectrum, which is subsequently denoted as ${}_{muscle}S_{group,i}$. We then subjected the spectra to MCR-ALS decomposition using the MATLAB software *MCR-ALS GUI v4c* [4,5]. First, PCA was used to show that all spectra can be decomposed into the same two components. Therefore, the corresponding MCR decomposition yielded two spectral components $SC1$ and $SC2$ (i.e., two rows of the S^T matrix in Equation (S1)), weighted by the concentrations ${}_{muscle}c1_{group,i}$ and ${}_{muscle}c2_{group,i}$ (i.e., two columns of the C matrix in Equation (S1)) that explained more than 98.3% of the total variance, i.e.,

$$
\begin{gathered}
{}_{muscle}S_{group,i} \approx {}_{muscle}c1_{group,i} \cdot SC1 + {}_{muscle}c2_{group,i} \cdot SC2 \\
\text{for} \\
muscle \in \{gluteus\ maximus, gastrocnemius\} \\
group \in \{Y - NDM, Y - STZ - DM, O - NDM, O - \ HFD - DM\} \\
i \in \{1,2, \ldots 9\}
\end{gathered}
\quad \text{(S2)}
$$

The second derivatives of the two MCR spectral components $SC1$ and $SC2$ were used to identify the vibrational frequency bands and their assignment to the macromolecular constituents of muscle tissue. The $c1$ and $c2$ concentrations (weights) were further elaborated statistically to characterize the skeletal muscles composition of the studied groups.

References

1. Worsfold, P.; Townshend, A.; Poole, C.F.; Miró, M. *Encyclopedia of Analytical Science*; Elsevier: Amsterdam, The Netherlands, 2019.
2. Lawton, W.H.; Sylvestre, E.A. Self Modeling Curve Resolution. *Technometrics* **1971**, *13*, 617–633. doi:10.1080/00401706.1971.10488823.
3. Ben-Amotz, D. Hydration-Shell Vibrational Spectroscopy. *J. Am. Chem. Soc.* **2019**, *141*, 10569–10580, doi:10.1021/jacs.9b02742.
4. MCR-ALS GUI V4c Available online: https://www.umu.se/en/research/infrastructure/visp/downloads/ (accessed on 18 November 2020).
5. Gorzsás, A. Vibrational Spectroscopy Core Facility Available online: https://www.umu.se/en/research/infrastructure/visp/downloads/ (accessed on 21 October 2020).
